# Carboxylation Reactions Using Carbon Dioxide as the C1 Source via Catalytically Generated Allyl Metal Intermediates

**DOI:** 10.3389/fchem.2019.00430

**Published:** 2019-06-27

**Authors:** Tetsuaki Fujihara, Yasushi Tsuji

**Affiliations:** Department of Energy and Hydrocarbon Chemistry, Graduate School of Engineering, Kyoto University, Kyoto, Japan

**Keywords:** carbon dioxide, carboxylic acids, allyl metals, transmetalation, oxidative addition, addition reactions

## Abstract

The use of carbon dioxide (CO_2_) is an important issue with regard to current climate research and the Earth's environment. Transition metal-catalyzed carboxylation reactions using CO_2_ are highly attractive. This review summarizes the transition metal-catalyzed carboxylation reactions of organic substrates with CO_2_ via allyl metal intermediates. First, carboxylation reactions via transmetalation are reviewed. Second, catalytic carboxylation reactions using allyl electrophiles and suitable reducing agents are summarized. The last section discusses the catalytic carboxylation reactions via addition reactions, affording allyl metal intermediates.

## Introduction

The development of fixation methods of carbon dioxide (CO_2_) is an important task for chemists (Aresta et al., 2014; Artz et al., 2018). However, CO_2_ is a kinetically and thermodynamically stable material. Therefore, using CO_2_ as a substrate poses several difficulties such as high-pressure or high-temperature reaction conditions. Transition metal-catalyzed carboxylation reactions of organic substrates with CO_2_ can allow the diverse production of carboxylic acids and their derivatives under mild reaction conditions via carbon-carbon bond formation. During the last decade, diverse transition metal-catalyzed carboxylation reactions have been reported and good reviews have also been published (Huang et al., 2011; Tsuji and Fujihara, 2012; Cai and Xie, 2013; Zhang and Hou, 2013; Liu et al., 2015; Yu et al., 2015; Borjesson et al., 2016; Sekine and Yamada, 2016; Wang et al., 2016; Hazari and Heimann, 2017; Chen et al., 2018; Luan and Ye, 2018; Tortajada et al., 2018).

**Scheme 1:**
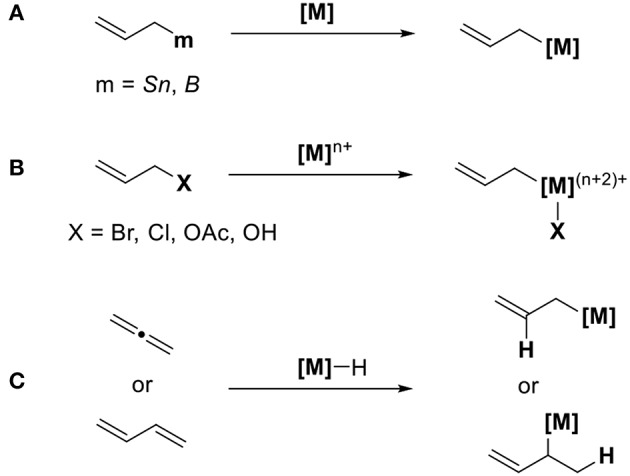
Generation of allyl metal intermediates: **(A)** transmetalation, **(B)** oxidative addition, and **(C)** addition reaction.

Allyl metal reagents are known for being excellent reagents during organic synthesis. The characteristic feature of allyl metal species is its isomerization via π-σ-π intermediates. σ-Allyl metal reagents act as nucleophiles, whereas π-allyl metals serve as electrophiles. When CO_2_ is used as electrophiles for transition metal-catalyzed reactions, a method to generate a catalytically active nucleophilic allyl metal intermediate is highly reliable. Scheme 1 shows three methods to access these allyl metal species from several organic substrates. The first method involves the transmetalation between allyl metal precursors such as allyl boranes or allylstannanes and transition metals (Scheme 1A). The second method involves the oxidative addition of allyl halides or allyl acetates using low-valent transition metals (Scheme 1B). In this case, suitable reducing agents must be used to regenerate low-valent active species. The third method involves the addition of metal species to conjugated dienes (Scheme 1C). In this reaction, the key is a regioselective addition that yields an ally metal intermediate selectively.

This paper overviews the transition metal-catalyzed carboxylation reactions of organic substrates with CO_2_ via allyl metal intermediates. First, Pd- and Cu-catalyzed carboxylation reactions via transmetalation are summarized. Second, Pd- and Ni-catalyzed carboxylation reactions using ally electrophiles such as allyl acetates are reviewed. Finally, the catalytic carboxylation reactions via addition reactions are discussed.

## Results and Discussion

### Transmetalation

**Scheme 2:**
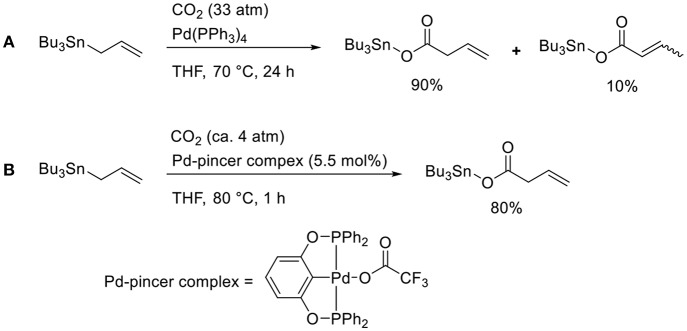
Catalytic carboxylation of allylstannane with CO_2_ using a Pd complex: **(A)** the reaction reported by Shi and Nicholas (1997) **(B)** the reaction reported by Johansson and Wendt (2007).

**Scheme 3:**
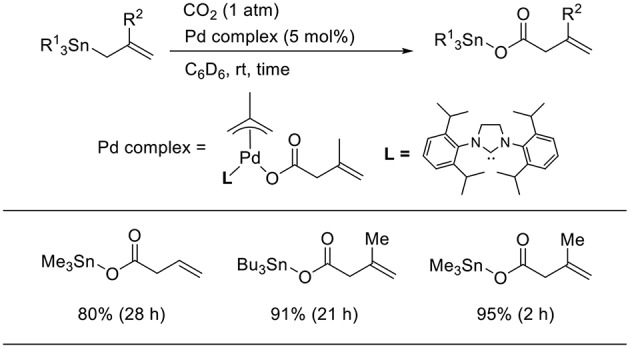
Catalytic carboxylation of allylstannane with CO_2_ using a Pd complex.

Transmetalation is a straightforward method that allows accessing catalytically active allyl metal intermediates during a carboxylation event. A pioneering work has been done by Nicholas who carried out the reaction of allylstannane using Pd(PPh_3_)_4_ as the catalyst in tetrahydrofurane (THF) at 70°C under 33 atm of CO_2_ (Scheme 2A, Shi and Nicholas, 1997). The reaction proceeds via allylstannane transmetalation, followed by a carboxylation reaction with CO_2_. Wendt observed an allyl Pd complex bearing a pincer ligand reacted with CO_2_ under mild reaction conditions (Scheme 2B, Johansson and Wendt, 2007). In 2011, Hazari reported the Pd-catalyzed carboxylation of allylstannanes and allyl boranes, which were used as substrates (Scheme 3, Wu and Hazari, 2011). The reactions proceeded using a Pd complex with an N-heterocyclic (NHC) ligand as the catalyst under 1 atm of CO_2_. Under the same reaction conditions, several allylboronates were also converted to the corresponding carboxylated products in good yields.

**Scheme 4:**
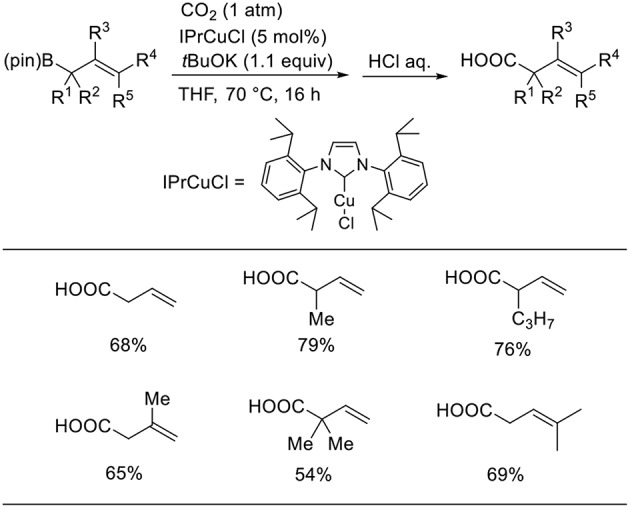
Cu-catalyzed carboxylation of allylboronates with CO_2_.

Duong reported the Cu-catalyzed carboxylation of allylboronic esters with CO_2_ (Scheme 4, Duong et al., 2013). The desired reactions proceeded in the presence of a Cu catalyst with IPr as the ligand in THF under 1 atm of CO_2_. Various allylboronic esters were subjected to the reaction, which generated corresponding products in moderate-to-high yields.

### Oxidative Addition

**Scheme 5:**
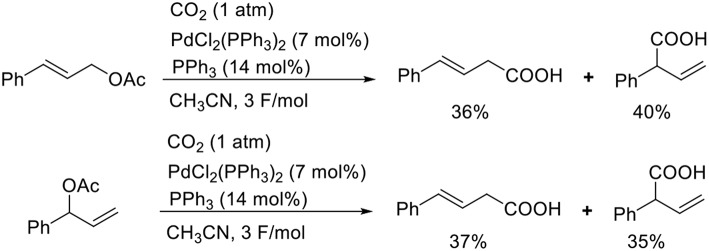
Pd-catalyzed carboxylation of cinnamyl acetate with CO_2_ under electrolysis.

Known as an elemental reaction during a Tsuji–Trost reaction, the oxidative addition of ally halides or allyl esters with low-valent metal species yields an ally metal intermediate (Trost and van Vranken, 1996; Trost and Crawley, 2003). Torii et al. reported the Pd-catalyzed carboxylation of allyl acetates when exposed to electroreductive reductions (Scheme 5, Torii et al., 1986). The reaction of cinnamyl acetate with CO_2_ in the presence of PdCl_2_(PPh_3_)_2_ and PPh_3_ afforded a mixture of regioisomers in 76% total yield. A branched substrate also afforded a mixture of regioisomers, indicating that the reaction proceeded via a π-allyl Pd intermediate. Dunach et al. found the Ni-catalyzed electrochemical carboxylation of allyl acetate with CO_2_. In the reaction, a mixture of two regioisomers was obtained (Medeiros et al., 2011). Mei et al. also found the Pd-catalyzed electrochemical carboxylation of allyl acetate, giving branched product selectively (Jiao et al., 2018).

**Scheme 6:**
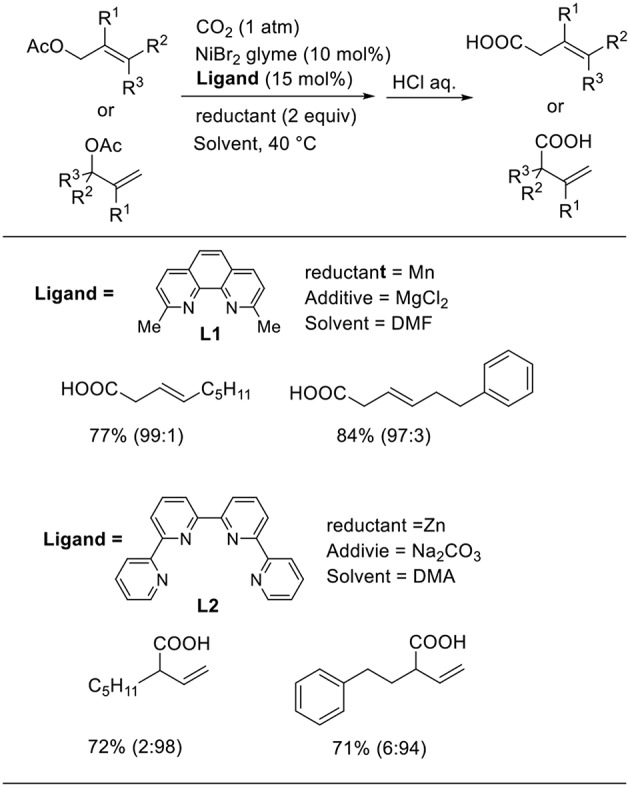
Ni-catalyzed ligand-controlled regiodivergent carboxylation of allyl acetates with CO_2_.

Martin et al. reported the Ni-catalyzed ligand-controlled regiodivergent carboxylation of allyl acetates using manganese as the reducing agent (Scheme 6, Moragas et al., 2014). When **L1** was used as the ligand, the linear carboxylated products were obtained. In contrast, the reactions using **L2** produced branched carboxylated products in good-to-high yields. The oxidative addition of a low-valent Ni species affords an allylic Ni intermediate. The reaction with CO_2_ yields Ni carboxylate intermediates; this is a regio-determining step for carboxylation.

**Scheme 7:**
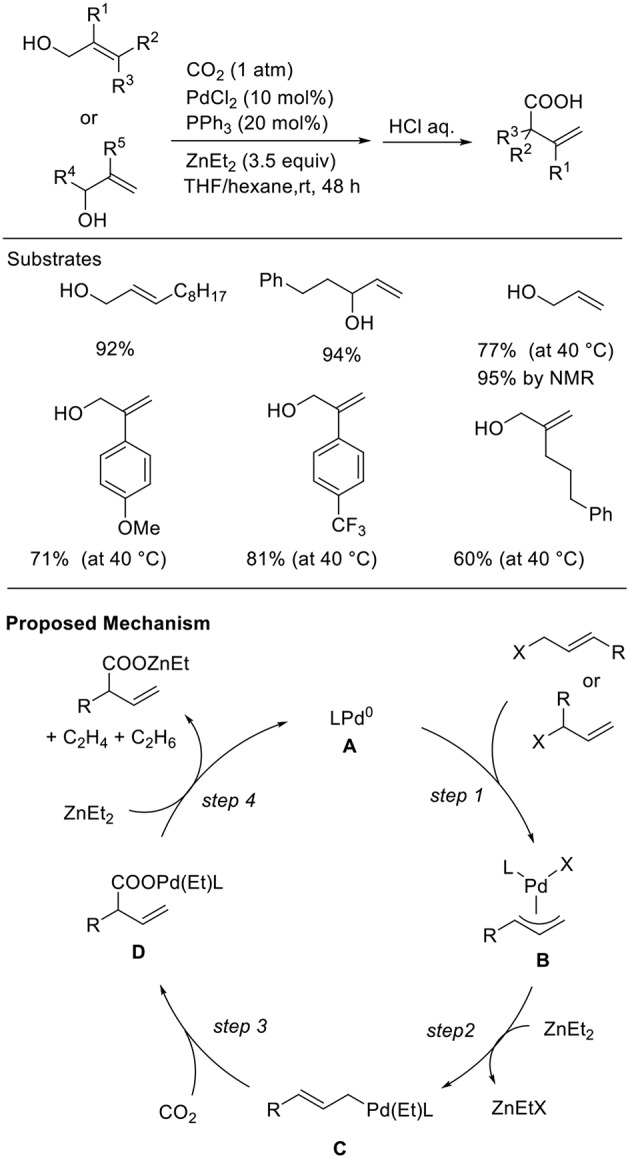
Pd-catalyzed carboxylation of allyl alcohols with CO_2_ using Et_2_Zn.

Allyl alcohols are excellent reagents that allow the generation of allyl metal intermediates. However, in general, the hydroxyl group must be converted to the corresponding halides or esters before their use. Thus, the direct use of alcohols as the substrate for carboxylation reactions is important for both atom- and step-economical points. Mita and Sato found the Pd-catalyzed carboxylation of allyl alcohols in the presence of Et_2_Zn (Scheme 7, Mita et al., 2015). This reaction proceeded in the presence of a Pd catalyst with a PPh_3_ ligand in a THF/hexane solution at room temperature under 1 atm of CO_2_. Diverse allyl alcohols were converted to the corresponding branched carboxylic acids in good to high yields with high regioselectivity. Noteworthy is that a wide range of functional groups such as an ester and a free hydroxyl group was tolerated in the reactions. Regarding a reaction mechanism, allylic alcohols would be activated by ZnEt_2_ followed by oxidative addition, leading to a π-allylpalladium intermediate (step 1). Then, nucleophilic σ-allylpalladium can capture CO_2_ to generate a carboxylato Pd intermediate (step 3). Finally, transmetalation followed by reduction can regenerate Pd(0) active species (step 4).

**Scheme 8:**
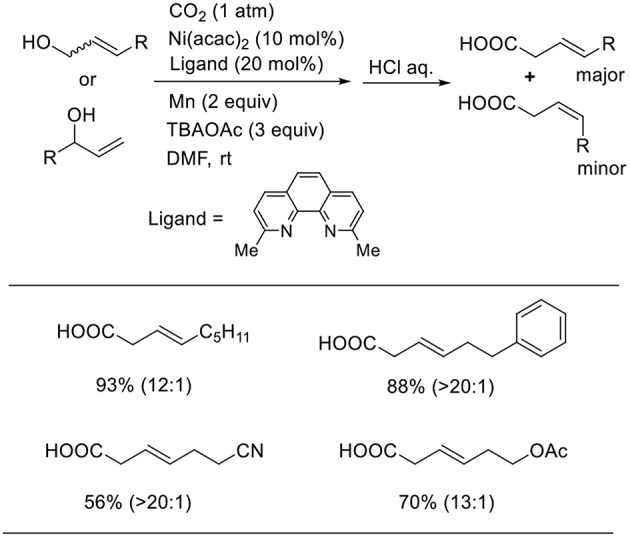
Ni-catalyzed carboxylation of allyl alcohols with CO_2_ using a manganese as reducing agent.

Mei also reported that a Ni complex catalyzed the carboxylation of allyl alcohols using manganese as a reducing agent (Scheme 8, Chen et al., 2017). This reaction proceeded in the presence of an Ni complex with a neocuproine ligand in N.N-dimethylformamide (DMF) at room temperature under 1 atm of CO_2_. In this reaction, tetrabutylammonium acetate (TBAOAc) was essential; without the additive, the desired product could not be obtained at all. The reactions using both linear and branched allyl alcohols produced linear carboxylic acids in good-to-high yields with high regioselectivity. The reactions tolerated several functionality such as cyano and ester groups.

**Scheme 9:**
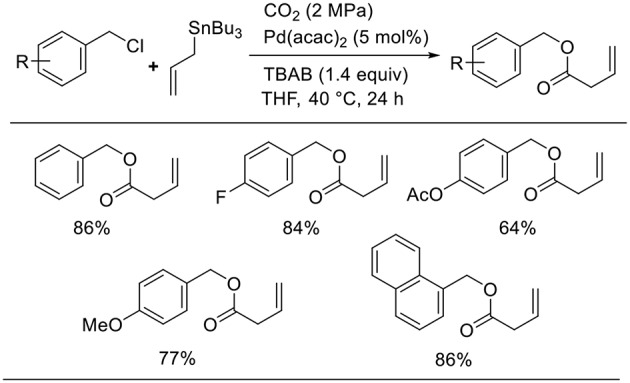
Pd-catalyzed carboxylative coupling of benzyl chlorides and an allylstannane under CO_2_.

Bao found the Pd-catalyzed carboxylative coupling of benzyl chlorides and an allylstannane under CO_2_ atmosphere (Scheme 9, Feng et al., 2013). Diverse benzyl chloride derivatives took part in the reactions, which produced the corresponding carboxylative coupling products in good-to-high yields. A key intermediate, the π-benzyl-π-allyl Pd complex, can be obtained by the oxidative addition of Pd(0) to benzyl chloride followed by transmetalation.

**Scheme 10:**
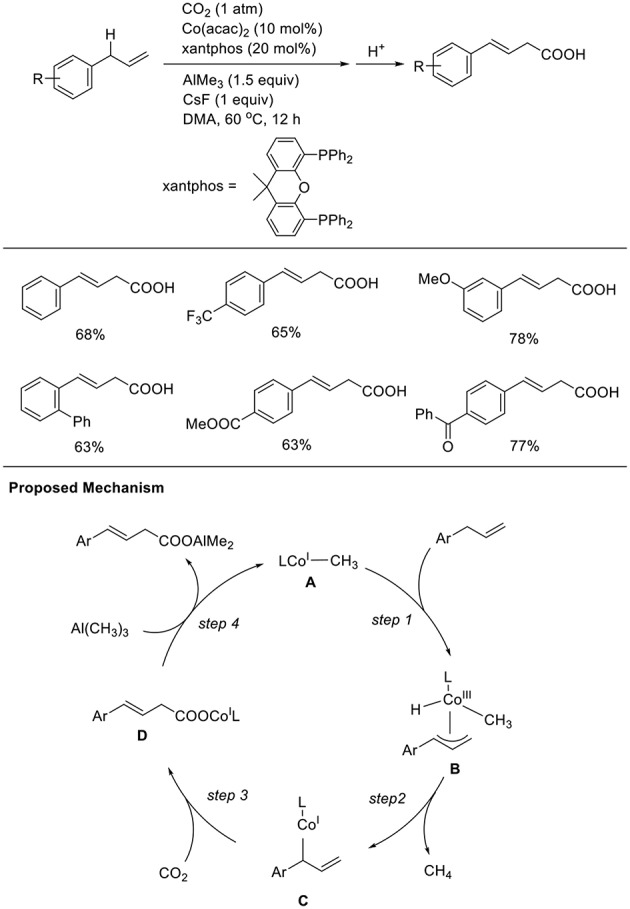
Co-catalyzed allylic C-H carboxylation of allylarenes and a proposed mechanism.

Transition metal-catalyzed carboxylation reaction via direct carbon-hydrogen (C-H) activation is a central research issue. Thus, the development of less reactive C-H carboxylation reactions is important. Mita and Sato observed a unique C-H carboxylation reaction with allylbenzene derivatives (Scheme 10, Michigami et al., 2017). The reaction of 4-phenyl allylbenzene was carried out in the presence of AlMe_3_ (3 equivalent) using Co(acac)_2_ and xantphos as the ligand in DMA at 60°C under 1 atm of CO_2_ in the presence of CsF as an additive. With these reaction conditions, a carboxylated product was obtained in 68% yield. Reactions were performed with diverse substrates, which produced corresponding carboxylated products in good-to-high yields. Notably, the reactions tolerated substrates having ester and ketone moieties, which are generally more reactive with nucleophiles than CO_2_. The reaction starts by generating a low-valent CH_3_-Co(I) species (**A**). The C-C double bond of the substrate coordinates to the cobalt center, and subsequent cleavage of the adjacent allylic C–H bond affords η^3^-allyl-Co(III) species **B** (step 1). Then, the reductive elimination of CH_4_ from **B** affords a low-valent allyl-Co(I) species **C** (step 2). Next, C–C bond formation at the γ-position occurs via a reaction with CO_2_, giving a carboxylato Co species **D** (step 3). Finally, a linear carboxylated product is obtained by the transmetalation between **D** and Al(CH_3_)_3_, with the concomitant regeneration of **A** (step 4).

### Addition Reactions

**Scheme 11:**
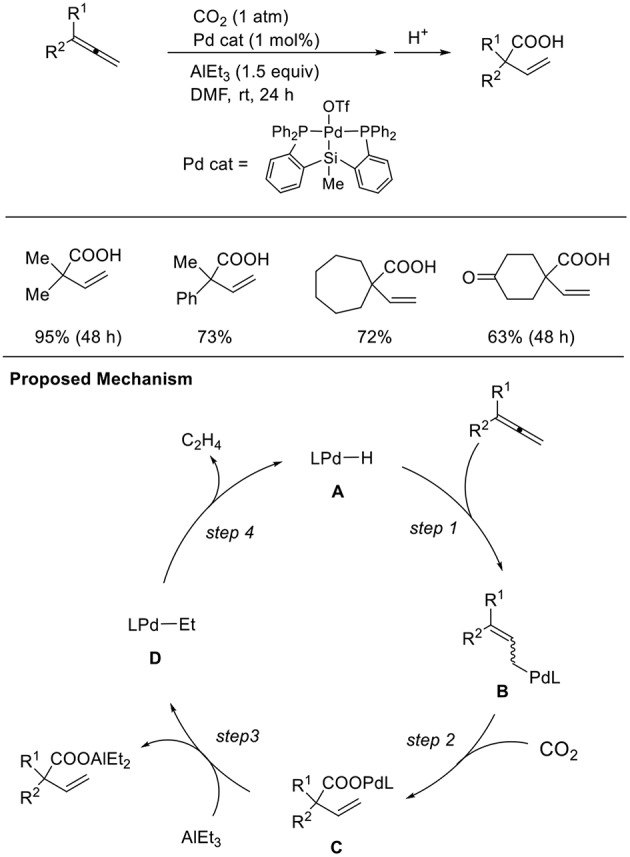
Pd-catalyzed hydrocarboxylation of 1,2-dienes and a proposed mechanism.

Addition reactions are an important method to generate nucleophilic metal intermediates using unsaturated hydrocarbons. Among them, 1,2-dienes as well as 1,3-dienes are candidates accessing allyl metal intermediates. However, the regioselectivity of addition reactions must be controlled. Iwasawa et al. found hydrocarboxylation of 1,2-dienes (Scheme 11, Takaya and Iwasawa, 2008). These reactions proceeded via the hydropalladation of Pd–H to 1,2-dienes (step 1) followed by carboxylation (step 2). A P–Si–P coordinated pincer ligand was crucial for the reaction and AlEt_3_ was employed as a reducing agent. With a similar Pd catalyst system, one-to-one coupling between 1,3-dienes and CO_2_ also occurred (Takaya et al., 2011). The reaction between a Pd precursor and triethylaluminum reagents followed by β-hydrogen elimination produces a Pd–H intermediate. Then, hydropalladation of conjugated dienes yields allyl palladium intermediates, which can regioselectively react with CO_2_.

**Scheme 12:**
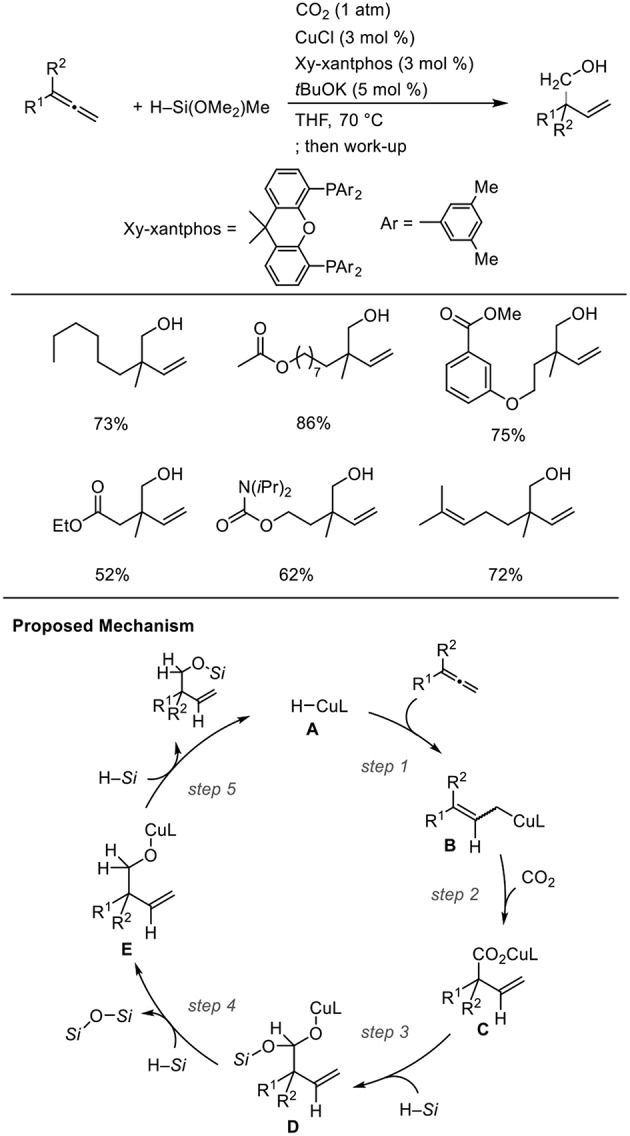
Cu-catalyzed enantioselective hydrohydroxymethylation of 1,2-dienes using a hydrosilane and CO_2_ and a proposed mechanism.

For Cu catalysts, we reported Cu-catalyzed hydrohydroxymethylation using 1,2-dienes as the substrate (Scheme 12, Tani et al., 2015). The reaction of 1,2-dienes with CO_2_ (1 atm) and a hydrosilane was examined in the presence of a copper catalyst bearing xantphos derivatives as the ligand. Using 1.5 mmol of hydrosilane, which corresponds to 2.0 equivalent for the required amount to yield the homoallyl alcohols, three H atoms were incorporated into the product. The corresponding homoallyl alcohols were obtained regioselectively, and the resulting terminal olefin moieties were preserved without hydrosilylation in the presence of an excess amount of hydrosilane. Various ester functionalities on the 1,2-dienes remained intact without reducing to their corresponding diols. Addition of a copper hydride **A** across a terminal double bond of allene **6** with the Cu atom at the less hindered site generates allylcopper intermediate **B** (step 1), which reacts with CO_2_ regioselectively at the γ-position via a six-membered transition state to afford a copper carboxylate **C** (step 2). Then, carboxylate **C** could be further reduced by the hydrosilane to copper alkoxide **E** via a silyl copper ketal derivative **D** (steps 3 and 4). Finally, σ-bond metathesis between **E** and the hydrosilane provides silyl ether and **A** regenerates (step 5).

**Scheme 13:**
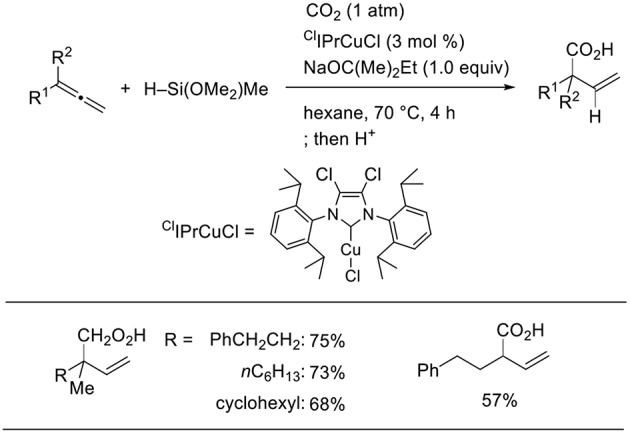
Cu-catalyzed hydrocarboxylation of 1,2-dienes.

Using a stoichiometric amount of base and ^Cl^IPr as the ligand, the product completely switched from homoallyl alcohols to β,γ-unsaturated carboxylic acids (Scheme 13, Tani et al., 2015). A stoichiometric amount of base is essential to induce selective carboxylic acid formation. Thus, products at two different oxidation levels (alcohols and carboxylic acids) were selectively prepared by simply adjusting the base equivalence and ligands in the Cu-catalyzed reactions.

**Scheme 14:**
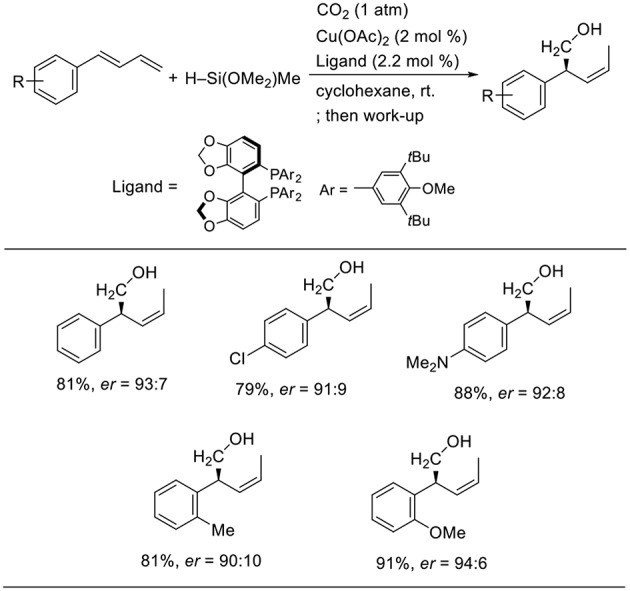
Cu-catalyzed enantioselective hydrohydroxymethylation of 1,3-dienes using a hydrosilane and CO_2_.

Yu reported enantioselective hydrohydroxymethylation of 1,3-dienes using a copper catalyst (Scheme 14, Gui et al., 2017). The reaction was carried out using 1-aryl-1,3-butadiens as the substrates and a hydrosilane as the reducing agent in cyclohexane at room temperature in the presence of a copper catalyst bearing 3,5-di-tert-butyl-4-methoxyphenyl (DTBM)-segphos as the ligand. Diverse 1-aryl-1,3-butadienes were used in the reactions, yielding the corresponding products in good-to-high yields with high enantioselectivity. The reactions tolerated several functional groups such as the chloro and amino groups.

**Scheme 15:**
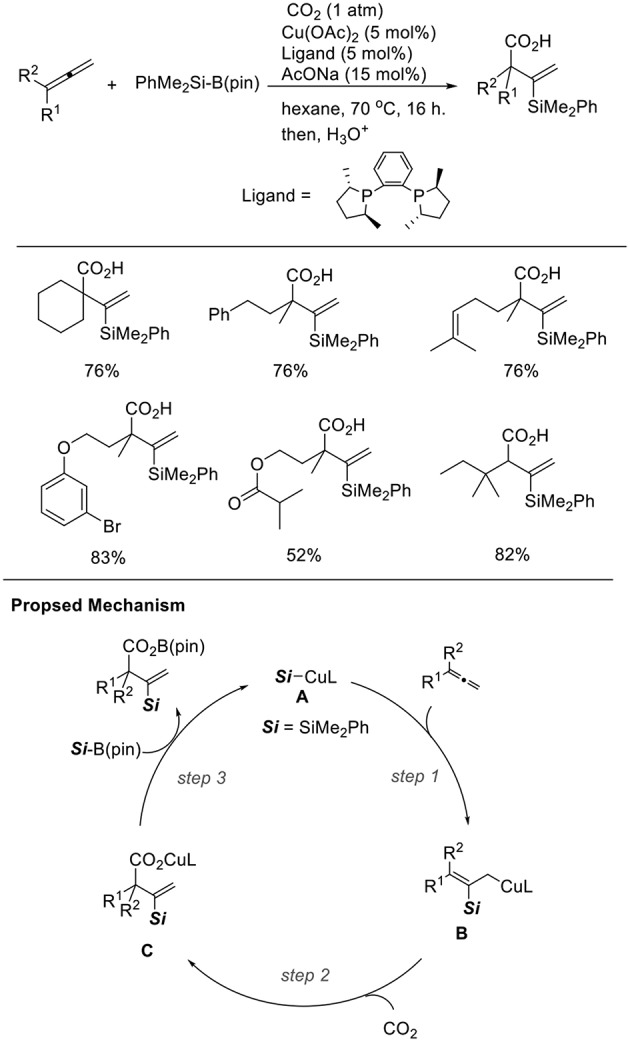
Cu-catalyzed silacarboxylation of 1,2-dienes and a proposed mechanism.

Silylcupration to carbon–carbon multiple bonds is a reliable and powerful process, which produces both C–Si and C–Cu bonds. We found the Cu-catalyzed silacarboxylation of 1,2-dienes (Scheme 15, Tani et al., 2014). The reaction was carried out by using 1,2-dienes and PhMe_2_Si–B(pin) under CO_2_ (1 atm) with a catalytic amount of Cu(OAc)_2_·H_2_O and *rac*-Me-DuPhos as the ligand. With these reaction conditions, diverse carboxylated vinylsilanes were regioselectively obtained in good yields. The functionalities, such as alkenyl and ester groups were tolerated under the reaction conditions. In the reactions, a β-silyl allyl copper intermediate (**B**) that was generated by the addition of silylcopper (**A**) across an allene (step 1) captured CO_2_ (step 2). Then, a carboxylate copper (**C**) reacts with a silylborane to afford **A** (step 3). The choice of an appropriate ligand can efficiently control the regioselectivity. Carboxylated allylsilanes were also obtained using CuCl/AcONa and PCy_3_ as the catalyst system.

### Conclusions

In conclusion, this short review summarized transition metal-catalyzed carboxylation reactions via allyl metal intermediates. Allylstannanes and allylboronates act as good reagents for Pd- or Cu-catalyzed carboxylation reactions via transmetalation. Transition metal-catalyzed allyl electrophiles via oxidative addition are disclosed herein. Ni complexes successfully produce ligand-controlled regiodivergent carboxylation. Allyl alcohols are possible substrates for Pd- and Ni-catalyzed carboxylation. Substrates, such as 1,2-dienes or 1,3-dienes, are usable for the generation of allyl metal intermediates via the addition of metal reagents. These processes involve hydrocarboxylation, hydrohydroxymethylation, and silacarboxylation. These methods allow efficient syntheses of unsaturated carboxylic acids using CO_2_ as the C1 source. In the future, these methodologies will be applied to synthesizing numerous valuable molecules in shorter steps using CO_2_.

## Author Contributions

TF arranged and wrote the manuscript. YT discussed the contents and commented on the manuscript.

### Conflict of Interest Statement

The authors declare that the research was conducted in the absence of any commercial or financial relationships that could be construed as a potential conflict of interest.
